# Health Literacy and Blood Glucose Level in Transitional Albania

**DOI:** 10.3389/fpubh.2020.00405

**Published:** 2020-08-18

**Authors:** Qamil Dika, Marsida Duli, Genc Burazeri, Dorina Toci, Helmut Brand, Ervin Toci

**Affiliations:** ^1^Department of Sports Medicine, University of Sports, Tirana, Albania; ^2^Faculty of Medicine, University of Medicine, Tirana, Albania; ^3^Department of International Health, School CAPHRI (Care and Public Health Research Institute), Maastricht University, Maastricht, Netherlands

**Keywords:** Albania, blood glucose level, diabetes, health literacy, knowledge

## Abstract

**Aim:** Our aim was to assess the independent association between blood glucose level and health literacy (HL) adjusting for many socio-demographic characteristics and body mass index (BMI) in an adult population in Albania, a transitional country in the South Eastern Europe.

**Methods:** A cross-sectional study was carried out in Tirana in 2012–2014 including a population-based sample of 1,154 individuals aged ≥18 years (57% women; mean age: 45.5 ± 16.4 years; response rate: 88.6%). HL was assessed by use of HLS-EU-Q instrument. Blood glucose level was measured in a fasting state by use of rapid finger stick method. Information on socio-demographic characteristics was collected, and BMI was calculated based on measurement of height and weight in all participants. General Linear Model (GLM) and binary logistic regression were used to assess the independent association of blood glucose level and HL adjusting for all socio-demographic factors and BMI.

**Results:** One-third of participants had pre-diabetes (100–125.9 mg/dl) and further 11% had diabetes (≥126 mg/dl) based on the measured blood glucose level. In fully-adjusted GLM, mean blood glucose level was significantly lower among individuals with excellent HL compared with their counterparts with inadequate HL (99.3 vs. 106.0, respectively). Furthermore, the odds for the presence of diabetes in the group of study participants whose HL was “inadequate” were 2.6 times higher (95% CI = 1.3–5.4) compared to those whose HL was “excellent.”

**Conclusion:** We obtained evidence of a strong and significant inverse relationship between measured blood glucose level and HL, independent of many socio-demographic characteristics and measured BMI in a population-based study in a country of the Western Balkans.

## Introduction

Health status is largely influenced by health behavior through complex processes involving multi-channel multi-layer interactions between individual socio-demographic factors and numerous external factors, influenced and modeled by cognitive skills and internal motivation and attitude toward certain behaviors (1, 2). Health literacy (HL), the ability to access, understand, appraise and apply health information in order to make appropriate health decisions (3), is often considered as a mediator in the processes leading to specific health behaviors and finally to health status (2, 4).

Maintaining the levels of blood glucose within normal range is important to health. As the levels of blood glucose fluctuate greatly during everyday life activities (5), health behavior becomes sensibly relevant for the prevention of diabetes in the general population (6) and especially important in the management of diabetes in terms of diet, physical activity, monitoring blood glucose levels, medication taking, promptly spotting the signs and symptoms of hypoglycemia and hyperglycemia and reducing glucose imbalance risks in general (7, 8). Among these, continuous professional and/or regular self-monitoring of glucose levels is critical for successful diabetes management (9, 10) and it is becoming increasingly relevant for the monitoring and prevention of diabetes among healthy and asymptomatic prediabetic population as well, based on recent developments predicting the expansion of use of glucose monitors across all population groups (11).

After the collapse of the communist regime in 1990, Albania is undergoing deep political and socioeconomic reforms which are also associated with changes in the epidemiological profile and health characteristics (12). According to the national Institute of Statistics (INSTAT), life expectancy in Albania in 2018 was 77.4 years in men and 80.5 years in women (13). In Albania, there is an increase of about 45% in the burden of non-communicable diseases (NCDs) for the period 1990–2017 (14). Yet, the age-standardized burden of NCDs (DALYs per 100,000 population) in Albania in 2017 was lower than in most of the former Yugoslavian Republics, excluding Slovenia (14).

Scientific research has reported significant inverse associations between HL and mean hemoglobin A1c (HbA1c) and mean plasma glucose levels, in both diabetic (15, 16) and general population (17), but the evidence about Albania and other transitional countries is scarce.

However, some other studies did not find significant associations of HL with plasma glucose levels and glycemic control (18, 19) and a very recent literature review concluded that the association of HL with glycemic control remains inconclusive (20).

In Albania and other Western Balkan countries there are no studies addressing comprehensively the association between HL and population fasting plasma glucose levels. A previous population-based study conducted in Albania has reported a significant association between HL and body mass index (BMI), irrespective of socio-demographic factors and socio-economic characteristics (21). Albania and other former communist countries in South Eastern Europe are heterogeneous in terms of within-society disease patterns and distribution of health characteristics and conventional risk factors (21). Therefore, it would be interesting to assess potential distinctive features including the magnitude of the relationship between HL and blood glucose level in these transitional societies. In this framework, the aim of our study was to shed light into the association of HL and glucose level in a population-based survey of adult men and women in Albania (21), a country in the Western Balkans which is currently undergoing tremendous changes and deep reforms in all sectors. More specifically, the objective of our analysis was to assess the independent association of HL and glucose level, adjusting for a full range of demographic factors and socioeconomic characteristics, as well as BMI. This analysis is based on the same study population of a previously conducted study including adult men and women in Albania, which was confined to assessment of the relationship between HL and BMI (21).

## Methods

### Study Design

This was a cross-sectional study carried out during the period September 2012—February 2014.

### Participants and Setting

The study population included a population-drawn simple random sample of 1,500 adult men and women (≥18 years), selected from the registries of family physicians operating in primary health care services of the city of Tirana, which is the capital of Albania. Further details about the study population and the sampling technique are reported elsewhere (21). Overall 1,154 individuals participated in the study (response rate: 88.6%) (21).

### Study Instruments

Data collection process included measurement of glucose level, BMI and administration of a structured questionnaire to all study participants.

### Observed Outcome

Glucose level was measured by use of a calibrated glucometer (finger stick method, a rapid glucose test kit). Glucose was measured in a fasting state in all study participants (22). In the analysis, normal glucose level was defined as <100 mg/dl, pre-diabetes as 100–125.9 mg/dl, and diabetes as ≥126 mg/dl (Table 1). These cut-off values are employed in primary health care in Albania for defining possible presence of pre-diabetes and diabetes (22).

**Table 1:** Distribution of blood glucose level among study participants.

**Variable**	**Value (mg/dl)**
**Glucose level**	
Mean	102.67
Standard deviation	28.76
Median	97
Interquartile Range	87–110
**Glucose level [*****n*** (%)]^*^	
Normal (<100 mg/dl)	478 (55.8)
Pre-diabetes (100–125.9 mg/dl)	285 (33.3)
Diabetes (≥126 mg/dl)	94 (11.0)
**Glucose level [*****n*** **(%)]**	
No diabetes	763 (89.1)
Diabetes	94 (11.0)

* *Information about glucose and/or other study variables is missing for 297 individuals*.

### Explanatory Factor

The original full version of the HLS-EU-Q instrument (3) was employed for assessment of HL among study participants. This tool was previously validated in Tirana in 2012 (22). The full version of HLS-EU-Q consists of 47 items measuring different HL dimensions that is access, understanding, appraisal and application of health information in the context of three specific domains: health care (16 items), disease prevention (16 items) and health promotion (15 items) (3).

Each of the HL items assessed the self-perceived difficulty of performing selected health-related tasks on a 4-point scale ranging from very easy (one) to very difficult (four) (3). The items' coding was reversed so that higher scores would indicate better HL (3). Subsequently, for each domain, a summary score was calculated based on scores of the respective items, and a general health index (comprising the scores of all 47 items) was also calculated. Next, the four resulting scores (general HL index, health care HL, disease prevention HL, and health promotion HL) were standardized on a scale ranging from 0 to 50 *[using the following formula: index* = *(mean–1)* ^*^50/3, where: “index” is the specified index calculated; “mean” is the average of all items for each individual; “1” is the minimal possible value for the mean (leading to a minimum value of the index of 0); “3” is the range of the mean; and “50” is the chosen maximum value of the new metric], to allow convenient calculations with indices and to simplify comparisons, in line with the recommendations and suggestions of the respective experts involved in the development of this instrument (3).

### Confounding Factors

Anthropometrics included weight (with precision of 100 g) which was measured in light clothes using a calibrated beam balance and height (with precision of 1 mm) which was measured using a tape attached to the wall with subjects not wearing shoes. BMI was calculated as weight (in kg) / height (in m^2^).

Furthermore, data on a full range of socio-demographic and socioeconomic characteristics was collected for all study participants, including age (in the analysis, categorized into: ≤25, 26–45, 46–65, and ≥66 years), sex (men vs. women), marital status (in the analysis, dichotomized into: married vs. single, divorced, and widowed), employment status (dichotomized into: unemployed vs. employed and/or retired), educational attainment (0–8, 9–12, and ≥13 years), economic status (trichotomized into: very bad/bad, average, and good/very good), and social status (low, middle, and high).

All participants signed an informed consent form after being explained the aims and procedures of the survey. The study was approved by the Albanian Committee of Bio-Medical Ethics on 23 July 2012.

### Data Analysis

For 297 individuals there was missing information on glucose level, BMI or other covariates. These cases were excluded from the analysis; hence, the statistical analysis consisted of 1,154–297 = 857 individuals.

Measures of central tendency and dispersion were calculated for the glucose level.

The chi-square test was used to compare the distribution of glucose levels (no diabetes vs. diabetes) according to socio-demographic characteristics (age, sex, marital status, employment, education, and economic and social status), BMI and HL of study participants.

General Linear Model was used to calculate mean values of glucose level among individuals distinguished by different HL categories (inadequate, problematic, sufficient and excellent). Initially, crude (unadjusted) mean values, their 95% confidence intervals (95%CIs) and *p*-values were calculated. Next, age-adjusted mean values, 95%CIs and *p*-values were calculated. Subsequently, general linear models were adjusted for all demographic characteristics (age, sex and marital status). Further adjustment consisted of controlling also for socioeconomic factors (education, employment, social status, and economic status). Finally, BMI was also additionally introduced into the general linear models. Multivariable-adjusted mean values of the glucose level, their respective 95%CIs and *p*-values were calculated.

In addition, binary logistic regression was used to assess the independent association of glucose level (dependent variable, dichotomized into: diabetes vs. no diabetes) with HL levels (inadequate, problematic, sufficient, and excellent). Crude (unadjusted) odds ratios (ORs), their respective 95% confidence intervals (95%CIs) and *p*-values were initially calculated. Next, logistic regression models were adjusted for age. Subsequently, all demographic characteristics (age, sex, and marital status) were entered into the logistic models. Subsequently, logistic regression models were additionally adjusted for socioeconomic factors (education, employment, social status, and economic status). Finally, BMI was also introduced into the logistic regression models. Multivariable-adjusted odds ratios ORs, their respective 95%CIs and *p*-values were calculated. All analyses met the goodness-of-fit criterion as appraised by the Hosmer-Lemeshow test.

In all cases, a *p*≤0.05 was considered as statistically significant.

Statistical Package for Social Sciences (SPSS, version 19.0) was used for all the statistical analyses.

## Results

### Description of the Study Group

On the whole, mean age in this study sample was 45.5 ± 16.4 years; 57% of participants were women; mean years of formal schooling were 12.6 years; about 82% of study participants perceived themselves as middle class, and about two-thirds (64%) reported an average economic status (21).

Overall, mean glucose level among study participants was 103±29 mg/dl (Table 1). Median value was 97 (interquartile range: 87–110). About one-third of participants exhibited pre-diabetes (100–125.9 mg/dl) upon measurement of glucose level and further 11% had diabetes (≥126 mg/dl).

Men had a higher prevalence of measured diabetes than women (13 vs. 10%), but this finding was not statistically significant (Table 2). The prevalence of diabetes, as expected, was considerably higher among older participants (≥66 years). There was evidence of a graded inverse relationship (although not statistically significant) of diabetes with educational level. Married participants displayed a significantly higher prevalence of diabetes, explained largely by the age difference with the non-married individuals. The prevalence of diabetes was lower among the better-off participants and those with a high social status. There was evidence of a graded relationship with BMI: the prevalence of diabetes was lowest among normal weight individuals (6%) and highest in obese participants (20%). Similarly, there was a graded relationship with HL levels: the prevalence of diabetes was lowest among participants with excellent HL (6%) and highest in those with inadequate HL (20%) (Table 2).

**Table 2:** Distribution of blood glucose level by socio-demographic characteristics, HL and BMI of study participants.

**Variable**	**Total**	**Diabetes status**		***P***
		**No diabetes**	**Diabetes**	
		**(*n* = 763)**	**(*n* = 94)**	
**Gender**				0.187^†^
Men	373 (43.5)^*^	326 (87.4)	47 (12.6)	
Women	484 (56.5)	437 (90.3)	47 (9.7)	
**Age-group**				<0.001
≤ 25 years	113 (13.2)	113 (100.0)	0 (–)	
26-45 years	249 (29.1)	241 (968)	8 (3.2)	
46-65 years	388 (45.3)	328 (84.5)	60 (15.5)	
≥66 years	107 (12.5)	81 (75.7)	26 (24.3)	
**Educational level**				0.139
0-8 years	125 (14.6)	107 (85.6)	18 (14.4)	
9-12 years	442 (51.6)	390 (88.2)	52 (11.8)	
≥13 years	290 (33.8)	266 (91.7)	24 (8.3)	
**Employment status**				0.257
Unemployed	160 (19.2)	145 (90.6)	15 (9.4)	
Employed and/or retired	672 (80.8)	594 (88.4)	78 (11.6)	
**Marital status**				0.008
Not married^‡^	293 (34.8)	272 (92.8)	21 (7.2)	
Married	549 (65.2)	477 (86.9)	72 (13.1)	
**Social status**				0.216
Low	89 (11.1)	80 (89.9)	9 (10.1)	
Middle	649 (81.2)	570 (57.8)	79 (12.2)	
High	61 (7.6)	58 (95.1)	3 (4.9)	
**Economic status**				0.047
Very bad/bad	106 (13.2)	90 (84.9)	16 (15.1)	
Average	527 (65.4)	463 (87.9)	64 (12.1)	
Good/very good	173 (21.5)	162 (93.6)	11 (6.4)	
**BMI**				<0.001
Normal	288 (33.6)	272 (94.4)	16 (5.6)	
Overweight	377 (44.0)	337 (89.4)	40 (10.6)	
Obese	192 (22.4)	154 (80.2)	38 (19.8)	
**Health literacy:**				<0.001
Inadequate	160 (20.0)	128 (80.0)	32 (20.0)	
Problematic	152 (19.0)	129 (84.9)	23 (15.1)	
Sufficient	213 (26.7)	190 (89.2)	23 (10.8)	
Excellent	274 (34.3)	259 (94.5)	15 (5.5)	

* *Absolute numbers and percentages in parentheses (row percentages for the glucose level categories, but column percentages for the totals). Discrepancies in the total numbers are due to the missing values*.

† *P-values from the chi-square test*.

‡ *Single, divorced and widowed*.

There was evidence of a mild positive linear association between glucose level and BMI (Spearman's rho = 0.27, *P* < 0.01), but a weak inverse correlation with HL (Spearman's rho = −0.15, *P* < 0.01) (not shown).

### Results of Univariate Analysis

Mean unadjusted glucose levels were significantly lower among participants with excellent HL levels compared with those with inadequate HL levels (98 vs. 111 mg/dl) (Table 3—model 1).

**Table 3:** Association of blood glucose level with HL (General Linear Models).

**Model**	Mean glucose^*^ (mg/dl)	95%CI^*^	P^*^
**Model 1**^‡^			**<0.001(3)**^†^
**Health literacy**			
Inadequate	110.9	106.4–115-5	<0.001
Problematic	105.9	101.2–110.5	0.006
Sufficient	102.2	98.3–106.1	0.095
Excellent	97.7	94.3–101.2	reference
**Model 2**^¶^			**0.065(3)**
**Health literacy**			
Inadequate	105.3	100.8–109.8	0.012
Problematic	103.1	98.5–107.7	0.069
Sufficient	101	97.1–104.9	0.222
Excellent	97.9	94.3–101.5	reference
**Model 3**^§^			**0.080(3)**
**Health literacy**
Inadequate	105.8	101.2–110.3	0.015
Problematic	103.6	98.9–108.2	0.086
Sufficient	101	97.1–105.0	0.347
Excellent	98.6	94.9–102.2	reference
Model 4^**^			**0.159(3)**
**Health literacy**			
Inadequate	105.2	99.4-111.0	0.034
Problematic	103.2	91.1-109.2	0.123
Sufficient	100.4	94.5-106.3	0.472
Excellent	98.4	92.9-103.7	reference
**Model 5**^††^			**0.191(3)**
**Health literacy**			
Inadequate	106	100.2–111.8	0.039
Problematic	103.7	97.6–109.7	0.161
Sufficient	101.1	95.2–107.0	0.514
Excellent	99.3	93.9–104.7	reference

* *Mean values, 95% confidence intervals (95%CI) and p-values from the General Linear Models*.

† *Overall p-value and degrees of freedom (in parentheses)*.

‡ *Model 1: crude (unadjusted) models*.

¶ *Model 2: age-adjusted models*.

§ *Model 3: adjusted for all demographic characteristics (age, sex and marital status)*.

** *Model 4: adjusted for all demographic characteristics and socioeconomic factors (education, employment, social status and economic status)*.

†† *Model 5: adjusted also for BMI*.

Conversely, in unadjusted binary logistic regression models (Table 4—model 1), the odds for the presence of diabetes in the group of study participants whose HL was “inadequate” were 4.3 times higher (95% CI = 2.3–8.3) compared to those whose HL was “excellent.”

**Table 4:** Association of HL with blood glucose level; multivariable-adjusted odds ratios (ORs: diabetes vs. no diabetes) from binary logistic regression.

**Model**	**OR**	**95%CI**	***P***
**Model 1**^†^			<0.001(3)^*^
**Health literacy**			
Inadequate	4.32	2.26-8.26	<0.001
Problematic	3.08	1.55-6.10	0.001
Sufficient	2.09	1.06-4.11	0.033
Excellent	1	Reference	-
**Model 2**^‡^			**0.027(3)**
**Health literacy**			
Inadequate	2.73	1.38-5.44	0.004
Problematic	2.44	1.21-4.95	0.013
Sufficient	1.88	0.94-3.77	0.074
Excellent	1	Reference	-
**Model 3**^¶^			**0.025(3)**
**Health literacy**			
Inadequate	2.77	1.39-5.49	0.004
Problematic	2.46	1.21-4.99	0.013
Sufficient	1.84	0.92-3.69	0.086
Excellent	1	Reference	-
**Model 4**^§^			**0.062(3)**
**Health literacy**			
Inadequate	2.6	1.26-5.37	0.010
Problematic	2.23	1.08-4.62	0.031
Sufficient	1.67	0.82-3.41	0.159
Excellent	1	Reference	-
Model 5^**^			**0.066(3)**
**Health literacy**			
Inadequate	2.62	1.26-5.44	0.01
Problematic	2.15	1.03-4.48	0.041
Sufficient	1.65	0.80-3.38	0.174
Excellent	1	Reference	-

* *Overall p-value and degrees of freedom (in parentheses)*.

† *Model 1: crude (unadjusted) models*.

‡ *Model 2: age-adjusted models*.

¶ *Model 3: adjusted for all demographic characteristics (age, sex, and marital status)*.

§ *Model 4: adjusted for all demographic characteristics and socioeconomic factors (education, employment, social status, and economic status)*.

** *Model 5: adjusted also for BMI*.

### Results of Multivariate Analysis

In the General Linear Models, adjustment for age (Table 3—model 2) and subsequently for all demographic factors (model 3) and further for socioeconomic characteristics (model 4), attenuated somehow the findings. In fully-adjusted models controlling for all socio-demographic factors and BMI (model 5), the difference between the two HL groups nevertheless persisted, with mean glucose levels being significantly lower among participants with excellent HL levels compared with those with inadequate HL levels (99 vs. 106 mg/dl). However, the association with “problematic” and “sufficient” HL categories was not statistically significant upon adjustment for all demographic factors, socioeconomic characteristics and BMI (model 5).

In age-adjusted binary logistic regression models (Table 4—model 2) the estimates were attenuated, whereas further adjustment for the other demographic factors (model 3) did not alter the findings. Additional adjustment for socioeconomic characteristics (model 4) slightly attenuated the estimates. In fully-adjusted models (Table 4—model 5), the odds for the presence of diabetes in the group of study participants whose HL was “inadequate” were 2.6 times higher (95% CI = 1.3–5.4) compared to those whose HL was “excellent.”

## Discussion

In this population-based sample of adult men and women in transitional Albania, we obtained evidence of a significant inverse association between measured blood glucose level and HL, which was assessed based on a well-established international instrument already validated in Albania (22). The inverse association between glucose level and HL was strong, consistent and persisted upon adjustment for a whole range of socio-demographic characteristics and measured BMI.

Our findings, in general, are in line with previous reports from international research.

A study among 228 individuals aged 30 years or older and seeking care at emergency department of a hospital in Georgetown, Guyana, reported that mean blood glucose level was higher among lower HL subjects (128.3 mg/dl) compared to high HL subjects (117.1 mg/dl), but the difference was not statistically significant (17). In our study we reported a lower overall mean glucose level (102.67 mg/dl). Nevertheless, the inverse association between HL and plasma glucose levels evidenced in our survey replicated rather similarly the finding of the Guyana study (17). In our survey, this association held true and significant even after controlling for potential confounding effects of basic socio-demographic and socioeconomic factors.

In a population-based study of elderly people aged 70–79 years, there was a reverse significant association between health literacy and mean fasting blood glucose among women, but not in men (23). Overall, these results are similar to those reported by our survey.

Our findings are also compatible with another population-based study including 1817 Japanese individuals (24).

Evidence of significant and inverse associations between health literacy and fasting plasma glucose is provided by another recent paper reporting on the associations of HL with an array of laboratory parameters among individuals aged 23–88 years receiving health checkup in Taipei, Taiwan (25).

Previous publications about this study population (among 1,154 individuals) in Albania have revealed inverse and significant associations of mean health literacy score with age, education and low social and economic status (21). Also, low HL individuals (inadequate and problematic HL) were about two times more likely to be overweight/obese compared to excellent HL participants in this population group (26). Findings from international literature also highlight such associations of HL with age, social status and economic status (3, 27) and overweight/obesity (28, 29). Since the proportion of prediabetic and diabetic individuals in our study was higher among these groups at high risk of low health literacy then we assume that all these factors play a role in the observed HL-glucose association.

Besides fasting plasma glucose levels we also reported about the prevalence of prediabetes (33.3%) and diabetes (11%) in this study population group. We found that diabetes prevalence (including prediabetes and diabetes individuals) was higher among married individuals and it was significantly and positively associated with age, but inversely associated with economic status, body mass index (BMI) and health literacy. Prediabetes is considered a high-risk condition for progression to full diabetes as one-quarter of affected persons will develop it in the next 5 years and more than two-thirds will do so in their lifetime (30). Prediabetes also increases the risk of diabetic complications and cardiovascular disease among those experiencing it compared to normal glucose level individuals (30).

According to Centers for Disease Control and Prevention (CDC) the prevalence of prediabetes among American adults aged ≥18 years was 33.9% in 2015 (31). The prevalence of prediabetes varies according to the definition (cut-offs) used (30). With regard to diabetes prevalence, it was 12.2% among US adults aged ≥18 years; diabetes prevalence increased with age and peaked at age ≥65 years with 25.2% (32). These prevalence rates are very similar to our findings. International Diabetes Federation reported that in 2019 the prevalence of diabetes in Albania among people aged 20–79 years was 9% with another 43% estimated to have undiagnosed diabetes (33). A study among 381 Saudi adult males aged 18–60 years reported that the prevalence of prediabetes and diabetes was 9.2 and 27.6%, respectively (34), illustrating the effect of using different cut-off values in the prevalence of these conditions.

Prevalence rate of prediabetes and diabetes vary according to different factors including education level, social and economic status, prevalence of overweight and obesity, access to healthcare services, etc. (30, 34, 35). We didn't find a significant gender difference regarding diabetes prevalence in our study even though the diabetes prevalence was higher in men than in women (12.6 vs. 9.7%, respectively), a finding not congruent with previous reports (36). On the other hand, women seem to be more aware about their diabetic status, more inclined to be treated and more likely to have controlled glycemic level compared to men according to a large study among individuals aged ≥18 years China (36). This finding is also supported by previous reports about the same population group in Albania suggesting that women exhibit higher proportions of excellent HL compared to men, possibly due to the higher and more frequent contacts of women with the health care system and their higher engagement in family issues (26). These elements could play a role in the lower prevalence of diabetes among women in Albania and could merit further scientific pursuit.

In our study population we reported that being married was significantly associated with a higher prevalence of diabetes, a finding supported by international research (34). Another study reported a higher prevalence of diabetes among married individuals only in crude unadjusted analysis but the association turned non-significant upon simultaneous controlling of confound effects (37). A large study among adults aged ≥18 years in Florida also reported that the prevalence of diabetes was significantly higher among married individuals in crude analysis but not in multivariable adjusted models (35). The authors of this paper also provided a conceptual model representing significant predictors of prediabetes and diabetes based on their findings, generally stating that age and sex affect diabetes directly and indirectly through mixed and parallel effects on income level, physical activity, BMI, various health conditions such as hypertension, arthritis and hypercholesterolemia all finally contributing to prediabetes and diabetes; furthermore, each of these factors, besides age and sex, also interact with each-other and in addition they affect directly the development of prediabetes and or diabetes (35).

The association of inadequate HL and diabetes is also supported by international literature (38, 39), with limited health literacy being quite prevalent about diabetic patients. A systematic review reported that the prevalence of limited health literacy among type 2 diabetes patients varied from about 7% in Switzerland to 82% in Taiwan (40). Basically, health literacy affects health outcomes (including diabetes outcomes) through acquisition of new knowledge, positive attitudes, greater self-efficacy and behavior change, obviously depending on individual reading fluency and prior knowledge and affected by prevailing cultural norms (39). In general low health literacy among diabetic patients is associated with worse diabetes knowledge, worse self-efficacy and self-care behaviors, worse glycemic control, increased risk of hypoglycemic events, increased likelihood of diabetes complications, etc. (38, 39).

Given the unfavorable position of low HL diabetic patients, it is necessary to pinpoint strategies and interventions that would help overcome this barrier. Such efforts include specifically tailored toolkits, guides or use of new online technologies intended to facilitate patient-provider communication (39). Other interventions could target health system and health institutions in order to modify them and facilitate their interaction of low health literacy patients (41). Education-based strategies, training about management of blood pressure and glucose-lowering medication, are also shown to improve diabetes outcomes among patients with limited health literacy (38).

Policymakers, decision makers and health professionals need to be aware about these effective and beneficial strategies, the application of which could facilitate the interaction of low health literacy patients and individuals with the health system.

There are some potential limitations of this study including the possibility of selection bias, information bias, timeline of the study and its cross-sectional design. On the whole, the response rate of this study was high (89%), but for 297 individuals there was no valid information on covariates including blood glucose level. In addition, at best, findings from this study can be generalized to the adult population of Tirana only given the fact that the survey was confined to this region. Blood glucose level and BMI were objectively assessed in all participants, which is reassuring. However, one measurement is not sufficient to establish the diagnosis of diabetes and, usually, other parameters are used (2-h Oral Glucose Tolerance Test [OGTT] and HbA1c testing)—posing another limitation of this study. Also, the instrument for assessment of HL was based on a well-developed and standardized tool (3) previously validated in the Albanian context (22). On the face of it, there is no reason to assume a differential reporting of HL between individuals with diabetes and their counterparts without diabetes. Yet, the possibility of information bias cannot be ruled out completely, particularly for the self-reported socioeconomic factors. Of note, the data used for the current analysis is more than 5 years old, which is another limitation of this study. Lastly, cross-sectional associations are not assumed to be causal and, therefore, the relationship between blood glucose level and HL should be more vigorously determined in future prospective studies.

In conclusion, regardless of these potential limitations, we obtained evidence of a strong and significant inverse relationship between measured blood glucose level and HL, independent of many socio-demographic characteristics and measured BMI in a population-based sample of adult men and women in a post-communist country.

In the context of current uncertainty prevailing in the international literature about the relationship between HL and blood glucose level, the present survey conducted in transitional Albania adds to the body of literature that supports a significant association between HL and fasting plasma glucose levels.

## Data Availability Statement

The datasets generated for this study are available on request to the corresponding author.

## Ethics Statement

The studies involving human participants were reviewed and approved by Albanian Committee of Bio-Medical Ethics. The patients/participants provided their written informed consent to participate in this study.

## Author Contributions

GB, HB, and ET contributed to the study conceptualization and design, analysis, and interpretation of the data. QD wrote the first draft of the article. MD and DT commented comprehensively on the manuscript. All authors have read and approved the submitted manuscript.

## Conflict of Interest

The authors declare that the research was conducted in the absence of any commercial or financial relationships that could be construed as a potential conflict of interest. The reviewer TS declared a past co-authorship with one of the authors GB to the handling Editor.
